# Synthesis of tunable copolymers of 3-hydroxybutyrate and 3-hydroxyvalerate by engineered *Halomonas bluephagenesis* and their characterizations

**DOI:** 10.1016/j.synbio.2025.11.007

**Published:** 2025-11-27

**Authors:** Shaowei Li, Jinghui Wang, Yaoyao Zhang, Kaixin Du, Jiangnan Chen, Jianping Sun, Huan Wang, Pengfei Ouyang, Xuanming Xu, Fuqing Wu, Fang Yang, Guo-Qiang Chen

**Affiliations:** a School of Life Sciences, Tsinghua University, Beijing, 100084, China; b PhaBuilder Biotechnology Co. Ltd., Shunyi District, Beijing, 101309, China; c Center for Synthetic and Systems Biology, Tsinghua University, Beijing, 100084, China; d MOE Key Lab of Industrial Biocatalysts, Dept Chemical Engineering, Tsinghua University, Beijing, 100084, China; e Tsinghua-Peking Center for Life Sciences, Tsinghua University, Beijing, 100084, China; f State Key Lab for Green Biomanufacturing, Beijing, 100084, China

**Keywords:** *Halomonas*, PHA, PHB, 3-Hydroxyvalerate, Synthetic biology, Next generation industrial biotechnology

## Abstract

Poly(3-hydroxybutyrate-*co*-3-hydroxyvalerate) (PHBV) with a 0–30 mol% controllable range of 3HV ratios was produced by *Halomonas bluephagenesis* (*H. bluephagenesis*) and characterized. An endogenous plasmid containing *scpA* and *scpB* encoding methylmalonyl-CoA epimerase and methylmalonyl-CoA decarboxylase, respectively, redirects succinyl-CoA toward propionyl-CoA, enabling *de novo* PHBV synthesis with a 1.7 mol% 3HV. Deletion of *sdhE* and *prpC* encoding succinate dehydrogenase and 2-methylcitrate synthase, respectively, further enhanced the 3HV to 4 mol%. *H. bluephagenesis* GZ05 was engineered for late-phase-specific MreB (a cytoskeletal protein) degradation, enlarged intracellular PHBV granules for enhanced PHBV synthesis, and convenient downstream. A series of growth experiments was conducted in a 7 L bioreactor fed with valerate to produce PHBV with various 3HV molar ratios (2–27 mol%). A quantitative relationship between valerate concentration and the final 3HV molar ratio was established with an R^2^ = 0.9833, enabling precise control of the 3HV ratio in PHBV. *H. bluephagenesis* GZ05 was grown to 100 g L^−1^ cell dry weight (CDW) containing 73 wt% PHBV consisting of 1.6 mol% 3HV in a 5000 L bioreactor. Thermal analysis demonstrated enhanced flexibility with higher 3HV content in PHBV.

## Graphical abstract

**Figure:**
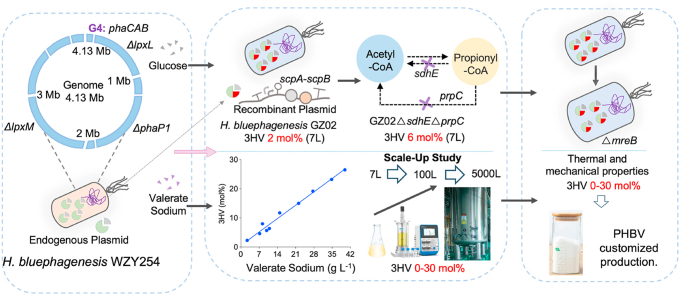

## 1 Introduction

Petroleum-based plastics are non-biodegradable, causing long-term environmental pollution and endangering living organisms, ultimately threatening the entire global ecosystem [[1], [2], [3], [4]]. Polyhydroxyalkanoates (PHA) are microbially produced biopolyesters that accumulate as intracellular carbon and energy reserves under nutrient-limited conditions [[5], [6], [7]]. PHA represent a promising class of eco-friendly bioplastics capable of reducing microplastic pollution and lowering emissions of carbon dioxide and other greenhouse gases [5,8,9].

Polyhydroxybutyrate (PHB) is the most common polyhydroxyalkanoate (PHA) with high crystallinity and hardness, but it suffers from brittleness, low elongation at break, and a narrow melt-processing window [[10], [11], [12], [13], [14]]. PHBV, composed of 3-hydroxybutyrate (3HB) and 3-hydroxyvalerate (3HV), exhibits improved thermal, mechanical, and physical properties compared to PHB, making it a highly promising biopolymer for diverse applications, including biomedical uses and petroleum-based plastic replacement [[15], [16], [17]]. The 3HV ratio in PHBV directly affects its degradation rate and thermodynamic properties, as demonstrated in prior studies [[18], [19], [20]]. However, the effect of varying 3HV ratios on PHBV properties has not been studied in great detail [20,21]. Investigating the influence of 3HV mol% on PHBV's final properties is crucial for its practical applications.

PHBV can be synthesized from glucose and related carbon sources by different microbial strains, such as the halophile *Halomonas* sp. YJ01, *Halomonas* sp. TD01, *Escherichia coli,* and *Corynebacterium glutamicum* WM001 et al., to produce variants with varying 3HV molar ratios [10,20,[22], [23], [24]]. The halotolerant *H. bluephagenesis* serves as a cornerstone chassis for NGIB, with its salt-adapted physiology enabling robust industrial-scale bioproduction under non-sterile conditions [25]. Compared with conventional microbial strains, this extremophile enables sterilization-free growth processes with over 35 % cost reduction [26]. Most importantly, extremophiles are easier to scale up compared to conventional microbes such as *Cupriavidus necator* or *Escherichia coli.* This will significantly reduce the cost of large-scale industrial production. After more than a decade of technological optimization and systematic engineering of PHA metabolic pathways, *H. bluephagenesis* has been successfully developed for industrial production of diverse PHA, including: native poly(3-hydroxybutyrate) (P3HB); poly(3-hydroxybutyrate-*co*-4-hydroxybutyrate) (P34HB); poly(3-hydroxybutyrate-*co*-3-hydroxyhexanoate) (PHBHHx); poly(3-hydroxybutyrate-*co*-5-hydroxyvalerate) (P3HB5HV); poly(3-hydroxybutyrate-*co*-3-hydroxyvalerate) (PHBV) and so on [20,22,[27], [28], [29], [30]].

In a previous study, PHBV was produced by *H. bluephagenesis* harboring *scpAB* operon encoding methylmalonyl-CoA mutase overexpression, and *the prpC* encoding 2-methylcitrate synthase knock-out and *sdhE* encoding succinate dehydrogenase assembly factor 2 knock-out, achieving an 18 mol% 3HV ratio in PHBV when cells were supplemented with glucose as the sole carbon source [22]. However, the CDW reached only 6 g L^−1^, and production has been reported only at the shake-flask level so far. When glucose is used as the sole carbon source for PHA synthesis, precise control of the 3HV molar ratio becomes challenging. Furthermore, the growth process exhibits poor stability in both optical density (OD_600_) and PHBV content. Engineered *H. bluephagenesis* achieved PHBV production with tunable 3HV ratios via metabolic engineering with valerate feeding, though property characterization was limited to select compositions [20]. Systematic optimization of valerate dosing to correlate 3HV content with PHBV material performance remains critical for application-specific PHBV design.

**Fig. 1:**
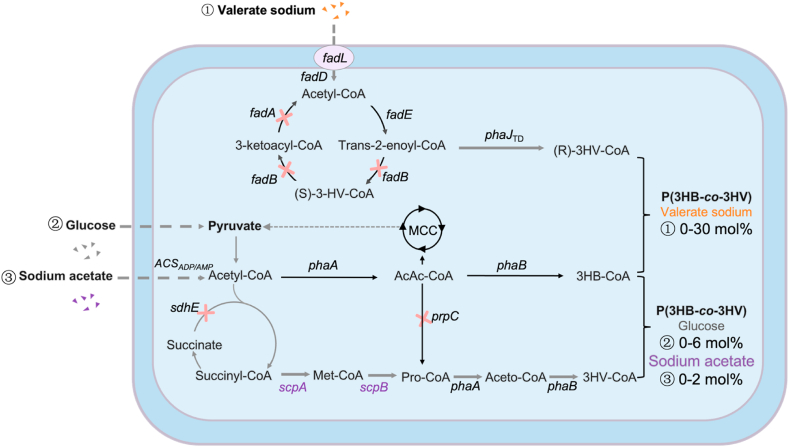
Engineering P(3HB-*co*-3HV) synthesis in *Halomonas bluephagenesis* grown in glucose or sodium acetate or mixed carbon sources of glucose and sodium valerate. Poly(3-hydroxybutyrate-*co*-3-hydroxyvalerate), abbreviated as P(3HB-*co*-3HV) or PHBV, is synthesized from glucose or sodium acetate or glucose and sodium valerate via three different metabolic pathways. For a related carbon source, glucose or sodium acetate supplies 3HB-CoA via *phaA* and *phaB* for 3HB monomer formation in PHBV, and the sodium valerate is converted to 3HV-CoA via *fadE* and *phaJ.* For an unrelated carbon source, glucose will be converted into 3HV-CoA under the catalysis of *scpA* and *scpB* via succinyl-CoA, methyl-malonyl-CoA, and propionyl-CoA. Enzymes encoded by genes: FadA, 3-ketoacyl-CoA thiolase; FadB, enoyl-CoA hydratase; FadE, acyl-CoA dehydrogenase; PhaJ_TD_, native enoyl-CoA-hydratase; PhaA, 3-ketothiolase; PhaB, NADH/NADPH-dependent acetoacetyl-CoA reductase; SdhE, succinate dehydrogenase assembly factor 2; ScpA, methyl-malonyl-CoA mutase; ScpB, methyl-malonyl-CoA decarboxylase; PrpC, 2-methylcitrate synthase; FadL, fatty acid transport protein; FadD, acyl-CoA synthetase; ACS_AMP_, the AMP-dependent acetyl-CoA synthetase; ACS_ADP_: the ADP-dependent acetyl-CoA synthetase.

This study aimed to develop metabolically engineered *H. bluephagenesis* strains for precise control and characterization of 3HV (0–27 mol%) in PHBV produced in a large bioreactor (Fig. 1). The *mreB* encoding a cytoskeletal protein was deleted to facilitate PHBV synthesis and downstream extraction for reduced production costs.

## 2 Materials and methods

### 2.1 Strains, plasmids construction, and culture conditions

*E*. *coli* S17-1 was used as a plasmid-constructed host strain and a conjugated donor strain [31]. *E. coli* S17-1 and its derivatives were cultured at 37 °C in Luria-Bertani (LB) medium. The LB medium contained 10 g L^−1^ NaCl (Analytical Reagent, Sinopharm Chemical Reagent Co., Ltd., China), 10 g L^−1^ tryptone (Analytical Reagent, Oxoid, England), and 5 g L^−1^ yeast extract (Analytical Reagent, Oxoid, England). *H. bluephagenesis* and its derivatives were cultured at 37 °C in Luria-Bertani 60 (60 LB) medium, which was composed of 60 g L^−1^ NaCl, 10 g L^−1^ tryptone, and 5 g L^−1^ yeast extract. Kanamycin (50 μg mL^−1^) [25,32], the antibiotics were from Macklin Biochemical Technology Co., Ltd (Shanghai, China). All strains used in this study are listed in Supplementary Table 1.

### 2.2 Plasmid construction and conjugation

For plasmid construction, the plasmid template was separated using the TIANprep Mini Plasmid Kit (Tiangen Biotech Co., Ltd., China). Then, the fragments for plasmid construction were generated via polymerase chain reaction (PCR) amplification by High-Fidelity DNA polymerase (New England Biolabs, USA) using the isolated plasmid template. The TIANquick Midi Purification Kit (Tiangen Biotech Co., Ltd., China) was used to purify the DNA after the gel electrophoresis separation. The plasmids were constructed by Gibson Assembly [33]. Single colonies screened by antibiotic plates were verified by colony-PCR and sequencing. The correct colonies were used in subsequent experiments, such as conjugation. Conjugation was conducted using the optimized protocol for *H. bluephagenesis* [27]. All plasmids and genes used in this study are listed in Table S1.

### 2.3 Gene editing via CRISPR/Cas9

Gene editing of *H. bluephagenesis* was conducted using the CRISPR/Cas9 method constructed for *H. bluephagenesis* [34]. Firstly, the gRNA-carrier plasmid was constructed by assembling two 1000-bp homologous arms, H1 and H2, flanking the gene knock-out targets. After verification by colony PCR and DNA sequencing, the plasmid was conjugated into *H. bluephagenesis* harboring a pQ08 plasmid with the *Cas9* protein. The right single colonies were selected and verified by PCR. Lastly, the plasmid was cured by culturing in LB medium without antibiotics for 5–7 growth cycles. The insertion of target genes is similar. All gene sequences edited in this study are listed in Table S2.

### 2.4 Shake-flask studies

60 LB or 60 MM medium containing different precursors is used for the growth of *H. bluephagenesis* and its derivatives. The 60 MM medium contains 6 % NaCl, 0.1 % (NH_4_)_2_SO_4_, 0.02 % MgSO_4_, 1.0 % Na_2_HPO_4_·12H_2_O, 0.15 % KH_2_PO_4_, 1.0 % trace element solution I, and 0.1 % trace element solution II [27]. Trace element solution I contains 0.5 % Fe (III)–NH_4_-citrate and 0.2 % CaCl_2_ without crystal water. Trace element solution II includes 0.01 % ZnSO_4_·7H_2_O, 0.003 % MnCl_2_·4H_2_O, 0.03 % H_3_BO_3_, 0.02 % CoCl_2_·6H_2_O, 0.001 % CuSO_4_·5H_2_O, 0.002 % NiCl_2_·6H_2_O and 0.003 % NaMoO_4_·2H_2_O. Before the shake flask experiment, the pH was adjusted to 8.5 using 5 M NaOH. Antibiotics were supplemented whenever necessary. For shake-flask studies, a single colony was picked and grown in 60 LB medium for 12 h to obtain the seed culture. Then this culture was transferred to fresh 60 LB medium at 1 % (v/v) inoculum ratio for a 12-h secondary seed cultivation. Finally, 2.5 mL (5 %) secondary seed culture was used to inoculate 50 mL 60 LB or 60 MM medium in the 500-mL shake-flask, then the medium system was cultured at 37 °C and 200 rpm for 48 h.

### 2.5 PHBV production in a 7-L or larger bioreactor

The lab-scale growth study was conducted in a 7-L fermenter (BioFlo 120, Eppendorf, Germany). The growth was conducted in a 3-L working volume bioreactor under non-sterile and open conditions. A single colony was used to inoculate the primary seed culture in 60 LB medium and transferred to fresh 60 LB medium at 1 % (v/v) inoculum ratio for a 12-h secondary seed culture until the OD_600_ reached 3–4. During the cultivation, the temperature and pH were maintained at 37 °C and 8.5. The dissolved oxygen (DO) concentration was maintained at 30 % and the agitation speed was initially set at 200 rpm until it was adjusted up to 800 rpm, coupled with DO. The air flow rate was maintained at 1 vvm (air volume/culture volume/min) during the fermentation.

The concentration of glucose was monitored by a SANNUO glucometer (GA-3, Sinocare Inc., China), and was manually maintained at around 10 g L^−1^ throughout the culture process, respectively. Corresponding valerate was added according to the expected 3HV molar ratio.

For growth in the 100 L bioreactor (Bailun, Beijing, China), the primary seed culture was prepared in the same manner as those for the shake flasks. Then 10 mL of primary seed culture was transformed to 1 L of fresh 60 LB in a 5 L conical flask and cultured at 37 °C and 200 rpm until the OD_600_ reached 3–5. Subsequently, 4 L of secondary seed culture was transferred to 41 L of fresh 60 MM in the 100 L bioreactor and cultured at 37 °C, pH 8.5 and 200 rpm. During the cultivation, the temperature and pH were maintained at 37 °C and 8.5, respectively. The dissolved oxygen (DO) concentration was maintained at 30 %. The concentration of glucose was manually maintained at around 10 g L^−1^ throughout the culture process. Related amount of valerate was added according to the expected 3HV molar ratio.

For growth in the 5000 L bioreactor (PhaBuilder, Beijing, China), the primary seed culture was prepared in the same manner as for the shake flask studies. Then, 10 mL of primary seed culture was transformed to 1 L of fresh 60 LB in a 5 L conical flask and cultured at 37 °C and 200 rpm until the OD_600_ reached 3–5. Subsequentely, 4 L of secondary seed culture was transferred to 296 L of fresh 60 MM in the 5000 L bioreactor and cultured at 37 °C, pH 8.5, and 200 rpm until the OD_600_ reached 5–6. Finally, the 300-L culture was transferred into the 5000-L bioreactor containing 2200 L of fresh 60 MM. During the cultivation, the temperature and pH were maintained at 37 °C and 8.5, respectively. The dissolved oxygen (DO) concentration was maintained at 30 %. The concentration of glucose was manually maintained at around 10 g L^−1^ throughout the culture process.

### 2.6 Studies on cell dry weights and PHA contents

After cultivation at 37 °C and 200 rpm for 48 h, bacterial cells were harvested by centrifugation at 10,000 rpm for 10 min and washed twice with the distilled water. Subsequently, the cells were freeze-dried at −80 °C, and then lyophilized for 12 h to achieve total dehydration. The lyophilized cell was weighed to determine the cell dry weight (CDW), and 30–40 mg of the lyophilized cell powder was subjected to an esterification reaction for 4 h at 100 °C. Subsequently, the PHA content and 3HV ratio were determined using gas chromatography (GC-2014, Shimadzu, Japan). Standards of 5–20 mg PHB (3HB unit standard; Boer Inc., China) or methyl 3-hydroxyvalerate (3HV unit standard; Sigma-Aldrich, USA) were used [4].

### 2.7 PHA extraction and purification

The intracellular PHA was extracted from the lyophilized cells using a Soxhlet extractor (Soxtec 2050, Foss, Denmark) following the protocol: boiled at 110 °C (3 h), eluted (2 h), and recycled (1 h). After the primary extraction, the PHBV was purified by dissolving it in chloroform and precipitating with 10-fold volume of cold absolute ethanol.

### 2.8 Gel permeation chromatography (GPC)

The molecular weights and polydispersities of the extracted PHBV were assayed by GPC (LC-20AD, Shimadzu, Japan) using a GPC-804C column equipped with a refractive index detector (RID). Before analysis, PHA samples were dissolved in chromatography-grade chloroform (2 mg mL^−1^) and filtered through a 0.22 μm nylon-membrane syringe filter (Jinglong, China). The injected sample volume was set to 40 μL, and chromatography-grade chloroform was used as the mobile phase at a flow rate of 1 mL min^−1^ at 40 °C. A series of polystyrene standards with average molar masses of 10, 20, 50, 100, 150, 300, 700, and 1000 kDa (Sigma-Aldrich, USA) were used to generate the calibration curve for the GPC analysis. All tests were conducted at room temperature (25 ± 0.1 °C).

### 2.9 Differential scanning calorimetry (DSC)

The thermal properties, including the glass transition temperature (T_g_) and melting temperature (T_m_), of the extracted PHA polymers were characterized by differential scanning calorimetry (DSC Q20, TA Instruments, USA). The analysis was performed under a nitrogen atmosphere. During the measurement, the nitrogen flow rate was 50 mL min^−1^, and PHA samples were cooled to −80 °C, then heated to 200 °C at a rate of 10 °C min^−1^. The PHA samples were quenched and heated again in the same program for a total of two cycles.

### 2.10 Mechanical property study

The extracted PHBV power was dried at 55–70 °C for 12 h. Then, standard samples (thickness: 4.0 ± 0.2 mm) were prepared for different tests using an injection molding machine: the standard tensile sample for test was prepared based on GB/T 1040.2–2022; the standard bending sample for test was prepared according to GB/T 9341-2008. Before the test, all samples were stored under the conditions stated in GB/T 2918 (23 ± 2 °C, 50 ± 10 % relative humidity) for 48 h to eliminate the residual stress caused by preparation. An Instron 3365 tensile-strength measurement device (Instron, USA) was used to conduct the stress-strain measurements with a stretch speed of 50 mm min^−1^ according to GB/T 1040–2018. The flexural strength was tested at a stretch speed of 3 mm min^−1^ based on GB/T 9341-2008, and the Notch impact strength was tested according to GB/T 1843-2008. Each sample was tested according to the protocol above at least three times in parallel, and the average values were reported as the tensile properties.

## 3 Results

### 3.1 De novo synthesis of PHBV by H. bluephagenesis encoding an endogenous plasmid

**Fig. 2:**
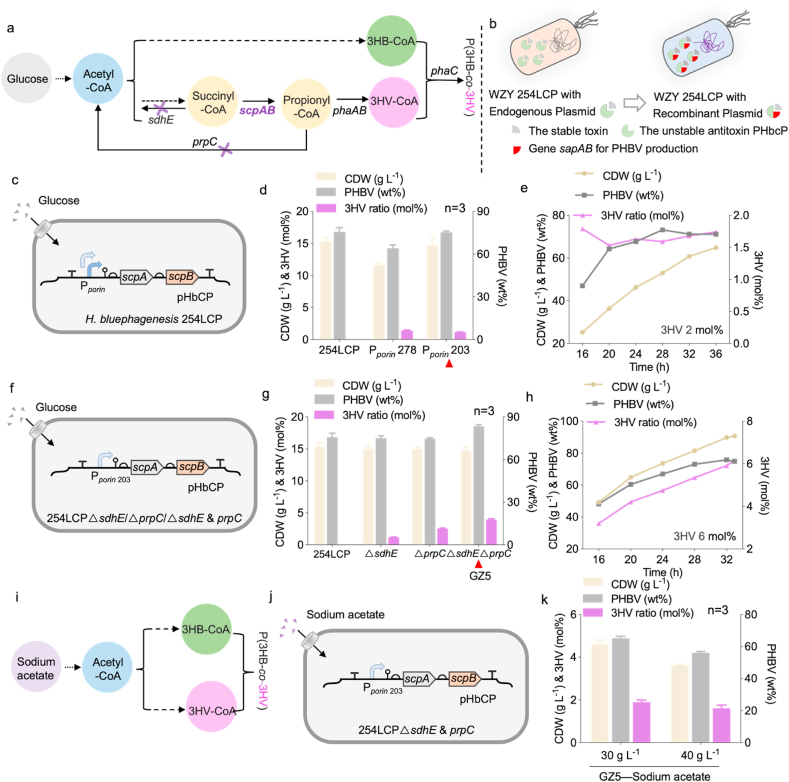
Effects of enhanced *scpAB* expression on PHBV synthesis by *H. bluephagensis* 254LCP and its derivatives harboring an endogenous plasmid. a) Engineered *de novo* synthesis pathway of PHBV in *Halomonas bluephagenesis* grown on glucose. b) Schematic diagram of the shake-flask and 7-L bioreactor studies. c) Schematic diagram of enhanced *scpAB* expression under the *porin* promoter encoded in the endogenous plasmid system in *H. bluephagenesis* 254LCP. d) Shake-flask studies for PHBV production by *H. bluephagenesis* 254LCP and its derivatives expressing the P_porin_-*scpAB* operon, shown in (c), cultured in 60 MM with 30 g L^−1^ glucose (n = 3). e) 7-L bioreactor studies for PHBV production by *H. bluephagenesis* 254LCP expressing the P_porin203_-*scpAB* operon. f) Schematic diagram of enhanced *scpAB* expression with P_porin203_ promoter in *H. bluephagenesis* 254LCP*ΔsdhE*/*ΔprpC*/*ΔsdhE* & *prpC* (n = 3)*.* g) Shake-flask studies for PHBV production by *H. bluephagenesis* 254LCP*ΔsdhE* & *prpC* expressing the P_porin203_-*scpAB* operon, shown in f), cultured in 60 MM with 30 g L^−1^ glucose. h) 7-L bioreactor studies for PHBV production by *H. bluephagenesis* 254LCP*ΔsdhE* & *prpC* expressing the P_porin203_-*scpAB* operon. i) Engineered PHBV synthesis pathway in *Halomonas bluephagenesis* grown on sodium acetate. j) The difference between j) and f) lies in the carbon source: sodium acetate was added for j), whereas glucose was added for f). k) Shake-flask studies for PHBV production by *H. bluephagenesis* 254LCP*ΔsdhE* & *prpC* expressing the P_porin203_-*scpAB* operon, shown in j), cultured in 60 MM with 30 g L^−1^ and 40 g L^−1^ sodium acetate as the sole carbon source (n = 3).

The endogenous plasmid offers high stability without antibiotic pressure while enabling efficient heterologous expression, providing a superior alternative to genome editing and conventional plasmid-based expression [35] (Fig. S1). Therefore, a PHBV *de novo* synthesis pathway was designed on an endogenous plasmid [35]. The introduction of the key enzymes *scpA* and *scpB* encoding methylmalonyl-CoA epimerase and methylmalonyl-CoA decarboxylase, respectively, which would convert succinyl-CoA to propionyl-CoA for 3HV formation (Fig. 2a). The effect of enhancing *scpA* and *scpB* expressions on the formation of 3HV was studied (Fig. 2).

Recombinant *H. bluephagenesis* 254LCP was used, with native *phaP1*, *LpxL,* and *LpxM* genes deleted from *H. bluephagenesis* TD01 [36]. This study demonstrates the successful production of PHBV using an engineered *H. bluephagenesis* 254LCP strain overexpressing the P_mmp1_-*scpA-scpB* gene cluster (Fig. S3b). To circumvent the detrimental impact of antibiotics and inducers on bacterial growth and their high cost, this study employed the industrially scalable *H. bluephagenesis* strain 254 to overexpress the endogenous plasmids pBhbCP-P_*porin*__203_-*scpA*-*scpB* and pBhbCP-P_*porin*__278_-*scpA-scpB*, respectively, (named as *H. bluephagenesis* GZ1 and GZ2) for PHBV production. *H. bluephagenesis* GZ2 exhibited higher PHBV content in shake-flask cultivation, achieving a cell dry weight (CDW) of 15 g L^−1^ containing 75 wt% PHBV consisting of 1.2 mol% 3HV (Fig. 2b-c). Consequently, *H. bluephagenesis* GZ2 was selected as the target strain for subsequent fermenter growth. In a 7L bioreactor, *H. bluephagenesis* GZ2 achieved a 65 g L^−1^ growth containing 71 wt% PHBV consisting of 2mol% 3HV after a 36h incubation.

However, propionyl-CoA is transformed into pyruvate via the methylcitrate cycle (MCC). The key enzyme catalyzing propionyl-CoA to pyruvate (*prpC*), encoding 2-methylcitrate synthase, was deleted to prevent the degradation of propionyl-CoA. At the same time, deletion of the *sdhE* encoding succinate dehydrogenase enhanced the metabolic flux. To enhance PHBV production, *sdhE*, *prpC*, or both *sdhE* and *prpC* were knocked out in *H. bluephagenesis* 254LCP (Fig. S3a), followed by overexpression of the pBhbC-P_*porin*__203_-*scpA-scpB*, generating *H. bluephagenesis* GZ3, GZ4, and GZ5, respectively. Shake-flask studies revealed that *H. bluephagenesis* GZ5 (Δ*sdhE*Δ*prpC* + operon overexpression) exhibited the best growth, achieving 15 g L^−1^ CDW containing 83 wt% PHBV consisting of 4 mol%3HV (Fig. 2a–b and 2f-2g). Subsequently, *H. bluephagenesis* GZ05 was selected for scaled-up production in a 7-L bioreactor. It was grown to 91 g L^−1^ CDW containing 75 wt% PHBV consisting of 6 mol% 3HV (Fig. 2h).

Additionally, to further decrease the cost of carbon source, we tried the production of PHBV with *H. bluephagenesis* GZ5 utilizing sodium acetate as the sole carbon source (Fig. 2i). The glucose was replaced with sodium acetate, which costs less yet has a higher theoretical carbon conversion rate (100 %) than glucose (66.7 %). Based on the previous result, we selected *H. bluephagenesis* GZ5 as the chassis for PHBV production with sodium acetate. The shake flask experiment was conducted to test the effect of sodium acetate as the sole carbon source. Shake-flask studies revealed that *H. bluephagenesis* GZ5 (Δ*sdhE*Δ*prpC* + operon overexpression) achieved 4.6 g L^−1^ CDW containing 65 wt% PHBV consisting of 1.9 mol%3HV when the sodium acetate concentration was 30 g L^−1^. However, when the concentration of sodium acetate increases to 40 g L^−1^, which is equivalent to the carbon molar number of 30 g L^−1^ glucose, the CDW (3.6 g L^−1^), PHA content (56 %), and 3HV molar ratio (1.6 mol%) were all obaerved decreased. This question resulted from the high pH caused by sodium acetate consumption, which may be solved during the 7-L bioreactor fermentation by controlling the pH at 8.5.

### 3.2 Morphology engineering via MreB deletion for enhanced PHBV synthesis

**Fig. 3:**
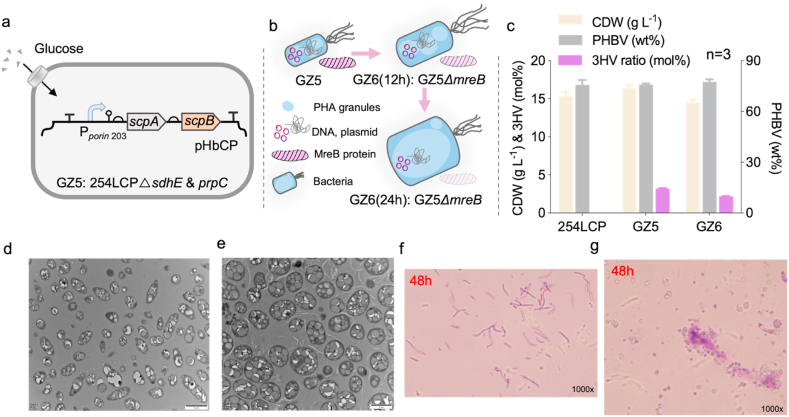
Morphology engineering and its effect on cell growth and PHA production. a) Schematic diagram of enhanced *scpAB* expression under P_porin203_ promoter in *H. bluephagenesis* 254LCP*ΔsdhE* & *prpC* (GZ5). b) Schematic diagram of enlarged *H. bluephagenesis* by deleting the *mreB* gene in GZ5 (*H. bluephagenesis* 254LCP*ΔsdhE* & *prpC*), named GZ6. c) Shake-flask studies for PHBV production by *H. bluephagenesis* 254LCP and its derivatives, *H. bluephagenesis* GZ5 and GZ6 (n = 3). d-e) Transmission electron microscope (TEM) results of *H. bluephagenesis* GZ5 and GZ6. f-g) Scanning electron microscope (SEM) results of *H. bluephagenesis* GZ5 and GZ6.

The gene *mreB*, encoding actin-like cytoskeletal proteins in bacteria, plays a critical role in maintaining cell shape [37]. It was deleted using the *sspB-ssrA* degradation system in *H. bluephagenesis* GZ5 for post-translational degradation to obtain larger spherical cells (Fig. 3a–b) [38]. Combined with overexpressing the native *phaAB* operon encoding acetoacetyl-CoA thiolase (PhaA) and acetoacetyl-CoA reductase (PhaB), the PHBV synthesis could be accelerated. However, the shake-flask growth showed similar results on the PHBV content from the cells overexpressing *phaAB* to that of the control group, with its CDW lower compared to the control; the 3HV molar ratio was only 2.2 %, a slight decrease compared to the control (Fig. 3c). Deletion of *mreB* significantly enlarged the cell size observed under transmission electron microscopy (TEM) and scanning electron microscopy (SEM) (Fig. 3d–g).

### 3.3 3HV ratios in PHBV synthesized by H. bluephagenesis fed with valerate

PHBV, consisting of different 3HV molar ratios, provides the PHBV material with a range of properties to satisfy the requirements of multiple applications. Yet the 3HV molar ratio of PHBV obtained from recombinant *H. bluephagenesis* grown on glucose as the sole carbon source was only 2–6 mol%, thus, sodium valerate was added to control the 3HV molar ratio in PHBV for cells grown on glucose [20]. Engineered *H. bluephagenesis* 254 was selected as it has been successfully used in a 400,000-L bioreactor for industrial PHBV production [36].

**Fig. 4:**
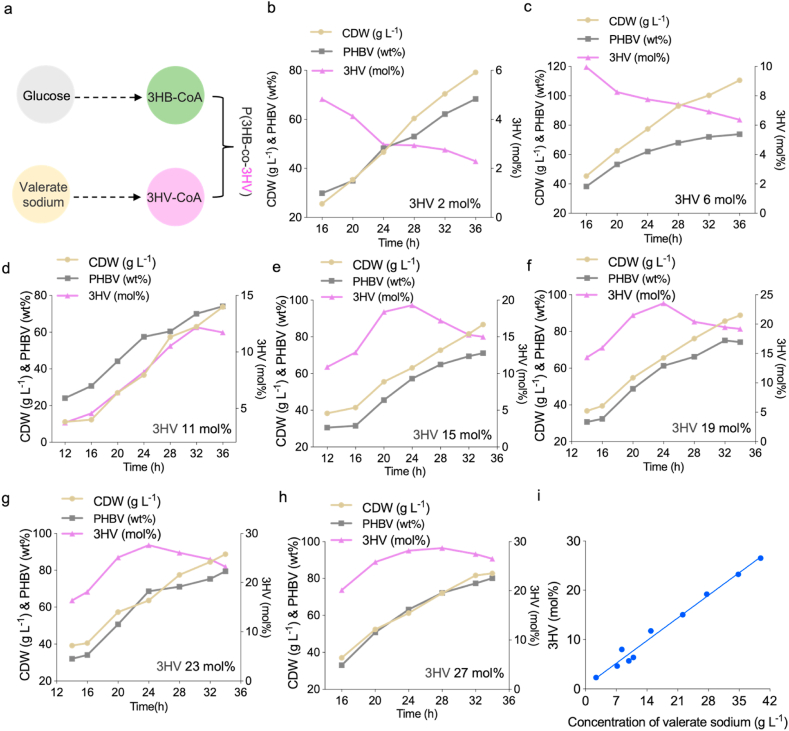
Production of PHBV with various 3HV ratios by engineered *H. bluephagenesis* 254 grown in a 7-L bioreactor fed with sodium valerate. a) PHBV synthesis pathway in *H. bluephagenesis* grown on glucose and valerate. b-h) Production of PHBV consisting of 2 %–27 % 3HV by the engineered *H. bluephagenesis* 254 grown in 7 L bioreactor containing 30 g L^−1^ glucose and various concentrations of valerate. i) Relationship between the concentration of valerate and the final 3HV molar ratio. The equation is y = 0.6555x+0.5995, x stands for the concentration of sodium valerate, y the final 3HV molar ratio.

Based on the shake flask studies, different concentrations of sodium valerate were fed to the cultures for production of various 3HV-molar ratios containing PHBV in a 7-L bioreactor (Fig. 4a). At 2.4 g L^−1^ sodium valerate, comparative *H. bluephagenesis* 254 was grown to 79.2 g L^−1^ of CDW containing 68.3 wt% PHBV consisting of 2.3 mol% 3HV molar fraction (Fig. 4b). Increasing sodium valerate feeding led to enhanced 3HV in PHBV up to 27 mol%. At 11 g L^−1^ sodium valerate, *H. bluephagenesis* 254 produced 111 g L^−1^ of CDW containing 74 wt% PHBV consisting of 6.4 mol% 3HV molar ratio (Fig. 4c). Compared to the 7-L bioreactor producing 2.3 mol% 3HV containing PHBV (Fig. 4b), feeding more sodium valerate moderately enhanced bacterial growth by 40 % in terms of CDW. At 15 g L^−1^ sodium valerate, *H. bluephagenesis* 254 was grown to 74 g L^−1^ containing 74 % PHBV consisting of 12 mol% 3HV (Fig. 4d). While at 22 g L^−1^ sodium valerate, *H. bluephagenesis* 254 produced 87 g L^−1^ CDW containing 71 wt% PHBV consisting of 15.02 mol% 3HV (Fig. 4e). *H. bluephagenesis* 254 achieved 89 g L^−1^ CDW containing 74 wt% PHBV consisting of 19 mol% 3HV at 28 g L^−1^ sodium valerate (Fig. 4f).

When sodium valerate was elevated to 35 g L^−1^, *H. bluephagenesis* 254 could be grown to 89 g L^−1^ containing 79 wt% PHBV consisting of 23 mol% 3HV (Fig. 4g).

The highest 27 mol% 3HV was achieved at 40 g L^−1^ sodium valerate, where *H. bluephagenesis* 254 was grown to 83 g L^−1^ CDW containing 80 wt% PHBV (Fig. 4h).

During the bioreactor studies, the fed sodium valerate was controlled at 0.4 g mL^−1^. Based on these results, the quantitative relationship between the end concentration of sodium valerate and the molar ratio of 3HV monomer was established with a very good linear correlation validated as R^2^ = 0.9833 (Fig. 4i).

As x represents the concentration of added sodium valerate and y the 3HV molar ratio, the relationship is y = 0.6555x + 0.5995. When sodium valerate is 12.59 g L^−1^ in the 7-L bioreactor, the 3HV molar ratio is approximately 8.8 % as suggested by the equation. Real bioreactor study showed that the molar ratio is 8.00 %, which is similar to the expectation of the equation (Fig. S4a). By adjusting the amount of added sodium valerate, the cells can produce PHBV with the expected 3HV molar ratio.

### 3.4 PHBV production by H. bluephagenesis 254 in 100-L and 5000-L bioreactors

**Fig. 5:**
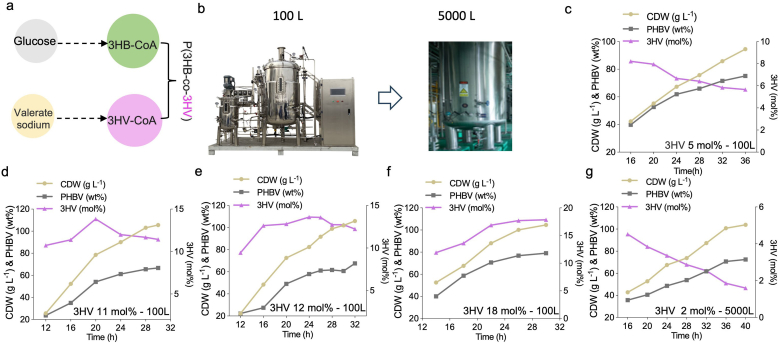
Production of PHBV consisting of expected 3HV molar ratios by engineered *H. bluephagenesis* 254 grown in 100-L and 5000-L bioreactors, respectively. a) *H. bluephagenesis* incubated in the 100-L and the 5000-L bioreactors, respectively. b-f) PHBV production with various 3HV molar ratios ranging from 5 to 18 mol% in the 100-L bioreactor containing glucose and various concentrations of valerate. g) PHBV production with 2 mol% 3HV molar ratio in the 5000-L bioreactor containing glucose and valerate.

Controllable 3HV molar ratio containing PHBV was successfully produced in the 7-L bioreactors with predictable results based on y = 0.6555x + 0.5995. The equation was tested in a 100 L bioreactor, aiming to produce PHBV containing approximately 5 mol%, 12 mol%, and 18 mol% 3HV in PHBV. It indicates that the equation in the 7-L bioreactor can be used to predict results in the 100-L bioreactor (Fig. 5a–f).

To further explore the scale-up potential of controllable 3HV in PHBV based on the equation, *H. bluephagenesis* 254 was grown in the 5000-L bioreactor for production of PHBV containing 2 mol% 3HV (Fig. 5g). Results showed a CDW over 100 g L^−1^ containing 72.5 wt% PHBV consisting of 1.6 mol% 3HV, an acceptable minor error.

### 3.5 Purification and characterization of PHBV containing various 3HV ratios

Bioreactor studies produced PHBV containing various 3HV molar ratios ranging from 2 % to 27 %; their molecular weights, thermal, and mechanical properties were studied (Table 1). The number-average (Mn), weight-average (Mw) molecular weights, and polydispersity index (PDI) show fluctuating changes with increasing 3HV content. For example, the Mw of P(3HB-*co*-2 mol%3HV) was 1302988 Da, while that of P(3HB-*co*-27 mol%3HV) decreased to 844046 Da. The PDI fluctuates between 2.0 and 2.28, reflecting the balance between chain growth and 3HV ratio during the polymerization process. Glass transition temperature (*T*_*g*_) decreased from 7.29 °C to −2.25 °C as the 3HV ratio increased. Similarly, crystallization temperature (*T*_*c*_) and melting temperature (*T*_*m*_) also showed a downward trend, indicating that 3HV interferes with the regular stacking of PHBV and reduces its crystallization ability. As expected, the tensile strength (TS) decreased from 34.75 MPa to 5.53 MPa, and the elongation at break (EAB) increased from 2.75 % to 9.03 %. The elastic modulus (E) and flexural strength (FS) generally decreased with the 3HV ratio increased, and the notch impact strength (NIS) and heat deflection temperature (HDT) also decreased with the increased 3HV content. These results show that the plasticizing effect of 3HV improves the elasticity but weakens the stiffness; the changing properties can be explored for various applications (Fig. S4b).

**Table 1:** Thermal and mechanical properties of various PHBV produced by *Halomonas bluephagenesis*.

Samples	Molecular Weight			Thermal properties			Mechanical properties					
	M_*w*_ (Da)	M_*n*_ (Da)	PDI	*T*_*g*_ (°C)	*T*_*c*_ (°C)	*T*_*m*_ (°C)	TS(MPa)	EAB (%)	E (MPa)	FS(MPa)	NIS(MPa)	HDT (°C)
P(3HB*-co*-2 mol%3HV)	1302988	681723	2.28	7.29	75.5	174.0	34.75 ± 2.19	2.75 ± 0.52	2119 ± 107	30.38 ± 2.27	2.48 ± 0.33	121.5 ± 4.9
P(3HB*-co*-6 mol%3HV)	901155	443095	2.16	4.00	77.3	171.7	30.02 ± 2.17	0.77 ± 0.19	5254.87 ± 235	23.59 ± 2.19	1.88 ± 0.29	124.2 ± 4.7
P(3HB*-co*-8 mol%3HV)	802621	397856	2.0	0.12	64.0	163.0	28.94 ± 1.97	1.23 ± 0.33	3473 ± 163	25.78 ± 2.23	1.72 ± 0.26	111.6 ± 4.8
P(3HB*-co*-11 mol%3HV)	767740	383507	2.0	−0.34	57.0	164.47	26.92 ± 1.92	1.97 ± 0.37	1713 ± 97	27.07 ± 2.21	1.97 ± 0.28	106.3 ± 4.7
P(3HB*-co*-15 mol%3HV)	881633	359897	2.13	−0.58	65.9	166.2	20.97 ± 1.68	3.2 ± 0.53	1716.35 ± 96	19.68 ± 2.01	3.9 ± 0.35	94.6 ± 4.2
P(3HB*-co*-19 mol%3HV)	877155	432277	2.25	−0.88	69.5	165.9	17.9 ± 1.45	3.34 ± 0.56	1186.66 ± 68	17.27 ± 1.90	2.1 ± 0.29	100.9 ± 4.4
P(3HB*-co*-23 mol%3HV)	871709	414042	2.01	−2.05	78.9	164.9	8.31 ± 0.76	8.8 ± 0.59	1035 ± 54	20 ± 2.07	4.6 ± 0.39	80.3 ± 4.3
P(3HB*-co*-27 mol%3HV)	844046	415832	2.11	−2.25	68.4	164.1	5.53 ± 0.42	9.03 ± 0.57	927 ± 46	16.65 ± 1.86	3.8 ± 0.36	75.4 ± 4.1

Note: Molecular weight: M_w_, weight average molecular weight; M_n_, number average molecular weight; PDI, polydispersity index (M_w_/M_n_). Thermal properties: T_g_, glass transition temperature; T_m_, melting temperature; T_c_, crystallization temperature. Mechanical properties: TS, tensile strength; EAB, elongation at break; E, elastic modulus; FS, flexural strength; NIS, notch impact strength. HDT, heat deflection temperature.

Better crystallization temperature, plasticity, and processability are important for making degradable forks, spoons, and chopsticks (Fig. S4b).

## 4 Conclusion

Engineered *H. bluephagenesis* 254LCP achieved *de-novo* PHBV synthesis from glucose via an endogenous plasmid encoding the 3HV synthesis pathway. 3HV was 2 mol% by expressing pHbCP-P_porin 203_-*scpAB*, it increased to 6 mol% after deleting *prpC/sdhE* in the cells grown in a 7 L bioreactor. Deletion of *mreB* enlarged cells without impairing growth or PHA synthesis. Up to 27 mol% 3HV in PHBV were produced fed with valerate. The established relationship between valerate and the 3HV ratio enabled predictable control for PHBV production. Increasing 3HV ratio progressively lowered thermal properties including *T*_*g*_*, T*_*c*_ and *T*_*m*_ of PHBV, tailoring crystallinity and mechanical properties for diverse applications.

## CRediT authorship contribution statement

**Shaowei Li:** Writing – original draft, Software, Methodology, Data curation, Conceptualization. **Jinghui Wang:** Data curation. **Yaoyao Zhang:** Validation, Data curation. **Kaixin Du:** Validation, Data curation. **Jiangnan Chen:** Validation, Investigation. **Jianping Sun:** Validation. **Huan Wang:** Validation. **Pengfei Ouyang:** Validation. **Xuanming Xu:** Validation. **Fuqing Wu:** Validation. **Fang Yang:** Writing – original draft, Visualization, Validation, Software, Methodology, Investigation, Formal analysis, Data curation, Conceptualization. **Guo-Qiang Chen:** Writing – review & editing, Writing – original draft, Validation, Supervision, Resources, Funding acquisition, Formal analysis, Data curation, Conceptualization.

## Declaration of competing interest

The author Guo-Qiang Chen is the Editor-in-Chief for Synthetic and Systems Biotechnology and was not involved in the editorial review or the decision to publish this article. The authors declare the following financial interests/personal relationships which may be considered as potential competing interests: Jinghui Wang, Yaoyao Zhang, Kaixin Du, Jianping Sun, Pengfei Ouyang, Xuanming Xu and Fang Yang are currently employed by PhaBuilder Biotechnology Co. Ltd. The research project is funded by PhaBuilder Co. Ltd.

## References

[1] Kotamraju A., Katakojwala R., Ramakrishna S., Venkata Mohan S. (2021). Low carbon biodegradable polymer matrices for sustainable future. Compos C Open Access.

[2] Andersson-Sköld Y., Johannesson M., Gustafsson M., Järlskog I., Lithner D., Polukarova M., Strömvall A.M. (2020). Microplastics from tyre and road wear A literature review.

[3] Singh N., Ogunseitan O.A., Wong M.H., Tang Y. (2022). Sustainable materials alternative to petrochemical plastics pollution: a review analysis. Sustain Horiz.

[4] Zhou M., Xu Y., Li J., Han Y., Yu H., Qi L., Chen J., Xiang H., Han J. (2025). Promoter engineering for enhanced poly(3-hydroxybutyrate-*co*-3-hydroxyvalerate) (PHBV) production in *Haloferax mediterranei*. Int J Biol Macromol.

[5] Hadri S.H., Tareen N., Hassan A., Naseer M., Ali K., Javed H. (2025). Alternatives to conventional plastics: polyhydroxyalkanoates (PHA) from microbial sources and recent approaches – a review. Process Saf Environ Prot.

[6] Koller M., Heeney D., Mukherjee A. (2025). Biodegradability of polyhydroxyalkanoate (PHA) biopolyesters in nature: a review. Biodegradation.

[7] Liu X., Park H., Ackermann Y.S., Avérous L., Ballerstedt H., Besenmatter W., Blázquez B., Bornscheuer U.T., Branson Y., Casey W., de Lorenzo V., Dong W., Floehr T., Godoy M.S., Ji Y., Jupke A., Klankermayer J., León D.S., Liu L., Liu X., Liu Y., Manoli M.T., Martínez-García E., Narancic T., Nogales J., O'Connor K., Osterthun O., Perrin R., Prieto M.A., Pollet E., Sarbu A., Schwaneberg U., Su H., Tang Z., Tiso T., Wang Z., Wei R., Welsing G., Wierckx N., Wolter B., Xiao G., Xing J., Zhao Y., Zhou J., Tan T., Blank L.M., Jiang M., Chen G.Q. (2025). Exploring biotechnology for plastic recycling, degradation and upcycling for a sustainable future. Biotechnol Adv.

[8] Acharjee S.A., Bharali P., Gogoi B., Sorhie V., Walling B. (2023). Alemtoshi. PHA-based bioplastic: a potential alternative to address microplastic pollution. Water Air Soil Pollut.

[9] Filonchyk M., Peterson M.P., Yan H., Gusev A., Zhang L., He Y., Yang S. (2024). Greenhouse gas emissions and reduction strategies for the world's largest greenhouse gas emitters. Sci Total Environ.

[10] Ma W., Zhou J., Cao Y., Wang J., Wang X. (2025). Insights into the microbial cell chassis design and engineering for production of poly(3-hydroxybutyrate-*co*-3-hydroxyvalerate). Food Biosci.

[11] Meixner K., Daffert C., Bauer L., Drosg B., Fritz I. (2022). PHB producing cyanobacteria found in the neighborhood-their isolation, purification and performance testing. Bioengineering.

[12] Najar I.N., Sharma P., Das R., Mondal K., Singh A.K., Tamang S., Hazra P., Thakur N., Bhanwaria R., Gandhi S.G., Kumar V. (2024). In search of poly-3-hydroxybutyrate (PHB): a comprehensive review unveiling applications and progress in fostering a sustainable bio-circular economy. Food Bioprod Process.

[13] Rajan K.P., Thomas S.P., Gopanna A., Chavali M. (2017).

[14] Yeo J.C.C., Muiruri J.K., Thitsartarn W., Li Z., He C. (2018). Recent advances in the development of biodegradable PHB-based toughening materials: approaches, advantages and applications. Mater Sci Eng C.

[15] Ibrahim M.I., Alsafadi D., Alamry K.A., Hussein M.A. (2021). Properties and applications of Poly(3-hydroxybutyrate-*co*-3-hydroxyvalerate) biocomposites. J Polym Environ.

[16] Jin A., Del Valle L.J., Puiggalí J. (2023). Copolymers and blends based on 3-Hydroxybutyrate and 3-Hydroxyvalerate units. Int J Mol Sci.

[17] Policastro G., Panico A., Fabbricino M. (2021). Improving biological production of poly(3-hydroxybutyrate-*co*-3-hydroxyvalerate) (PHBV) co-polymer: a critical review. Rev Environ Sci Biotechnol.

[18] Ferre-Guell A., Winterburn J. (2018). Biosynthesis and characterization of polyhydroxyalkanoates with controlled composition and microstructure. Biomacromolecules.

[19] Lyshtva P., Voronova V., Barbir J., Leal Filho W., Kröger S.D., Witt G., Miksch L., Saborowski R., Gutow L., Frank C., Emmerstorfer-Augustin A., Agustin-Salazar S., Cerruti P., Santagata G., Stagnaro P., D'Arrigo C., Vignolo M., Krång A.-S., Strömberg E., Lehtinen L., Annunen V. (2024). Degradation of a poly(3-hydroxybutyrate-*co*-3-hydroxyvalerate) (PHBV) compound in different environments. Heliyon.

[20] Wang H., Ouyang Y., Yang W., He H., Chen J., Yuan Y., Park H., Wu F., Yang F., Chen G.Q. (2025). Production and characterization of copolymers consisting of 3-hydroxybutyrate and increased 3-hydroxyvalerate by β-oxidation weakened *Halomonas*. Metab Eng.

[21] Shang L., Fei Q., Zhang Y.H., Wang X.Z., Fan D.-D., Chang H.N. (2012). Thermal properties and biodegradability studies of Poly(3-hydroxybutyrate-*co*-3-hydroxyvalerate). J Polym Environ.

[22] Chen Y., Chen X.Y., Du H.T., Zhang X., Ma Y.M., Chen J.C., Ye J.W., Jiang X.R., Chen G.Q. (2019). Chromosome engineering of the TCA cycle in Halomonas bluephagenesis for production of copolymers of 3-hydroxybutyrate and 3-hydroxyvalerate (PHBV). Metab Eng.

[23] Horng Y.T., Chien C.C., Huang C.T., Wei Y.H., Chen S.Y., Lan J.C.W., Soo P.C. (2013). Biosynthesis of poly(3-hydroxybutyrate-*co*-3-hydroxyvalerate) with co-expressed propionate permease (*prpP*), beta-ketothiolase B (*bktB*), and propionate-CoA synthase (*prpE*) in *Escherichia coli*. Biochem Eng J.

[24] Yin J., Yang J., Yu X., Chen T., He S. (2023). Enhanced poly(3-hydroxybutyrate-*co*-3-hydroxyvalerate) production from high-concentration propionate by a novel halophile *Halomonas sp*. YJ01: detoxification of the 2-methylcitrate cycle. Bioresour Technol.

[25] Yan X., Wang J., Wen R., Chen X., Chen G.Q. (2025). The halo of future bio-industry based on engineering *Halomonas*. Metab Eng.

[26] Ye J.W., Chen G.Q. (2021). *Halomonas* as a chassis. Essays Biochem.

[27] Tan D., Wu Q., Chen J.C., Chen G.Q. (2014). Engineering *Halomonas* TD01 for the low-cost production of polyhydroxyalkanoates. Metab Eng.

[28] Wang H., Ye J.W., Chen X., Yuan Y., Shi J., Liu X., Yang F., Ma Y., Chen J.C., Wu F., Lan Y., Wu Q., Tong Y., Chen G.Q. (2023). Production of PHA Copolymers consisting of 3-Hydroxybutyrate and 3-Hydroxyhexanoate (PHBHHx) by Recombinant *Halomonas bluephagenesis*. Chem Eng J.

[29] Yan X., Liu X., Yu L.P., Wu F., Jiang X.R., Chen G.Q. (2022). Biosynthesis of diverse α,ω-diol-derived polyhydroxyalkanoates by engineered *Halomonas bluephagenesis*. Metab Eng.

[30] Ye J., Hu D., Che X., Jiang X., Li T., Chen J., Zhang H.M., Chen G.Q. (2018). Engineering of *Halomonas bluephagenesis* for low cost production of poly(3-hydroxybutyrate-*co*-4-hydroxybutyrate) from glucose. Metab Eng.

[31] Simon R., Priefer U., Pühler A. (1983). A broad host range mobilization system for in vivo genetic engineering: Transposon mutagenesis in gram negative bacteria. Nat Biotechnol.

[32] Tang H., Gao J., Wang H., Sun M., Zhang S., Song C., Li Q. (2025). Characterization of the genome editing with miniature DNA nucleases *TnpB* and *IscB* in *Escherichia coli* strains. Commun Biol.

[33] Gibson D.G., Benders G.A., Andrews-Pfannkoch C., Denisova E.A., Baden-Tillson H., Zaveri J., Stockwell T.B., Brownley A., Thomas D.W., Algire M.A., Merryman C., Young L., Noskov V.N., Glass J.I., Venter J.C., Hutchison C.A., Smith H.O. (2008). Complete chemical synthesis, assembly, and cloning of a Mycoplasma genitalium genome. Science.

[34] Qin Q., Ling C., Zhao Y., Yang T., Yin J., Guo Y., Chen G.Q. (2018). CRISPR/Cas9 editing genome of extremophile *Halomonas spp*. Metab Eng.

[35] Ren K., Zhao Y., Chen G.Q., Ao X., Wu Q. (2024). Construction of a stable expression system based on the endogenous hbpB/hbpC toxin-antitoxin system of *Halomonas bluephagenesis*. ACS Synth Biol.

[36] Wang Z., Zheng Y., Ji M., Zhang X., Wang H., Chen Y., Wu Q., Chen G.Q. (2022). Hyperproduction of PHA copolymers containing high fractions of 4-hydroxybutyrate (4HB) by outer membrane-defected *Halomonas bluephagenesis* grown in bioreactors. Microb Biotechnol.

[37] Wang Y., jiang Y., Song Z., Zhu C., Tang Y., Peng J., MreB P. Liu (2025). Unraveling the molecular mechanisms of bacterial shape, division, and environmental adaptation. Cell Commun Signal.

[38] Chen Y.L., Liu X., Zhang L.Z., Yang J.S., Guo W.K., Zheng S., Wang J.L., Wu F.Q., Yan X., Wu Q., Chen G.Q. (2025). Cell sizes matter for industrial bioproduction, a case of polyhydroxybutyrate. Adv Sci.

