# Supplementary material for: Synthesis of tunable copolymers of 3-hydroxybutyrate and 3-hydroxyvalerate by engineered Halomonas bluephagenesis and their characterizations
Source: Synth Syst Biotechnol. 2025 Nov 27;12:91–100. doi: 10.1016/j.synbio.2025.11.007 (PMC12702219; doi:10.1016/j.synbio.2025.11.007)
Supplement: Multimedia component 1 [file mmc1.docx]

Supplementary info for Original research paper entitled:

**Synthesis of Tunable Copolymers of 3-Hydroxybutyrate and 3-Hydroxyvalerate by Engineered *Halomonas bluephagenesis* and Their Characterizations.**

**
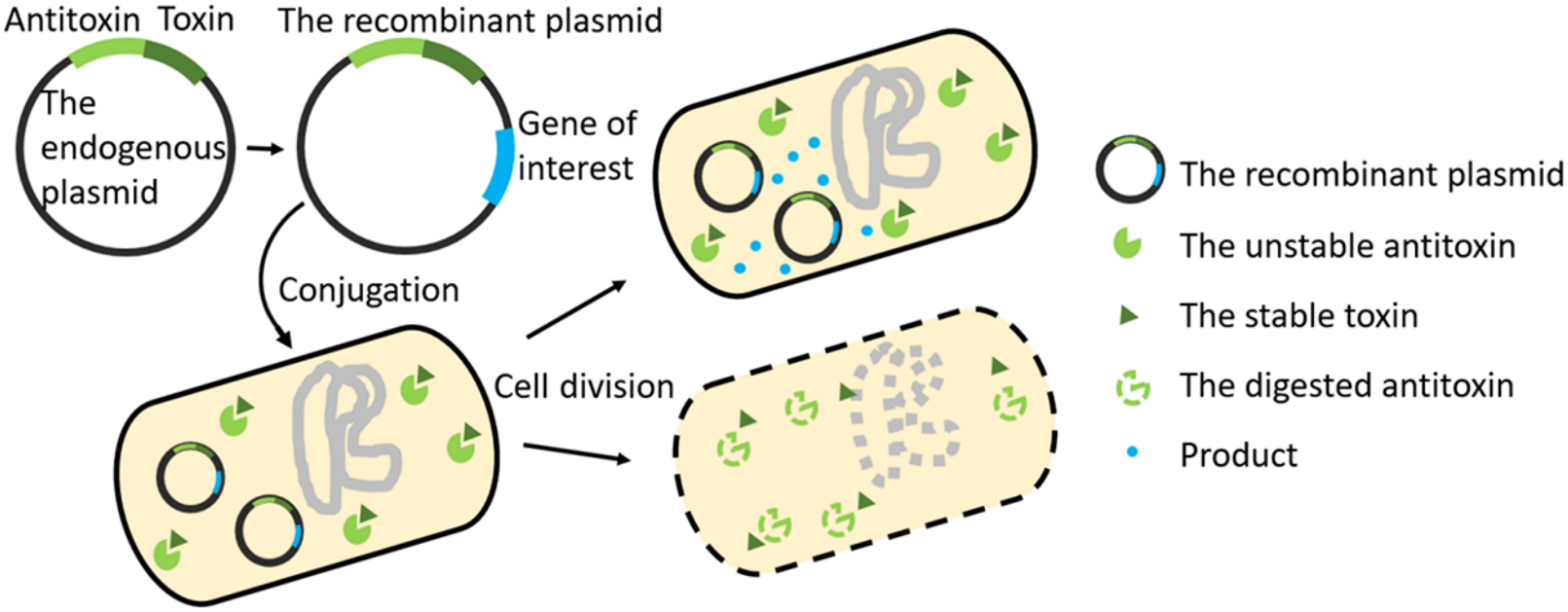
**

**Sup. Fig 1. Schematic diagram of the endogenous *hbpB/hbpC* toxin−antitoxin system of *Halomonas bluephagenesis^[1]^.***

The novel hbpB/hbpC toxin-antitoxin system enables high plasmid stability over 7 days of subculture without antibiotics in *H. bluephagenesis*. This tool facilitates efficient engineering of halophilic bacteria for next-generation industrial biotechnology (NGIB).

**
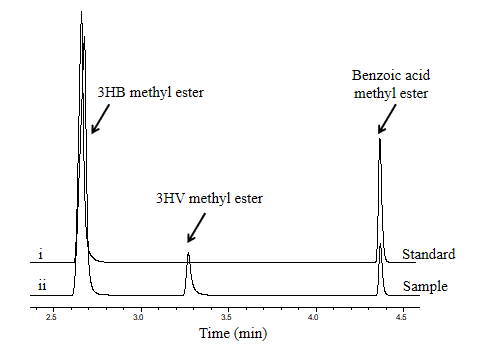
**

**Sup. Fig. 2 Gas chromatography (GC) analysis of PHBV samples.**

The GC band of 3HB methyl ester appears at approximately 2.65 min, while the 3HV 3.3 min.

**
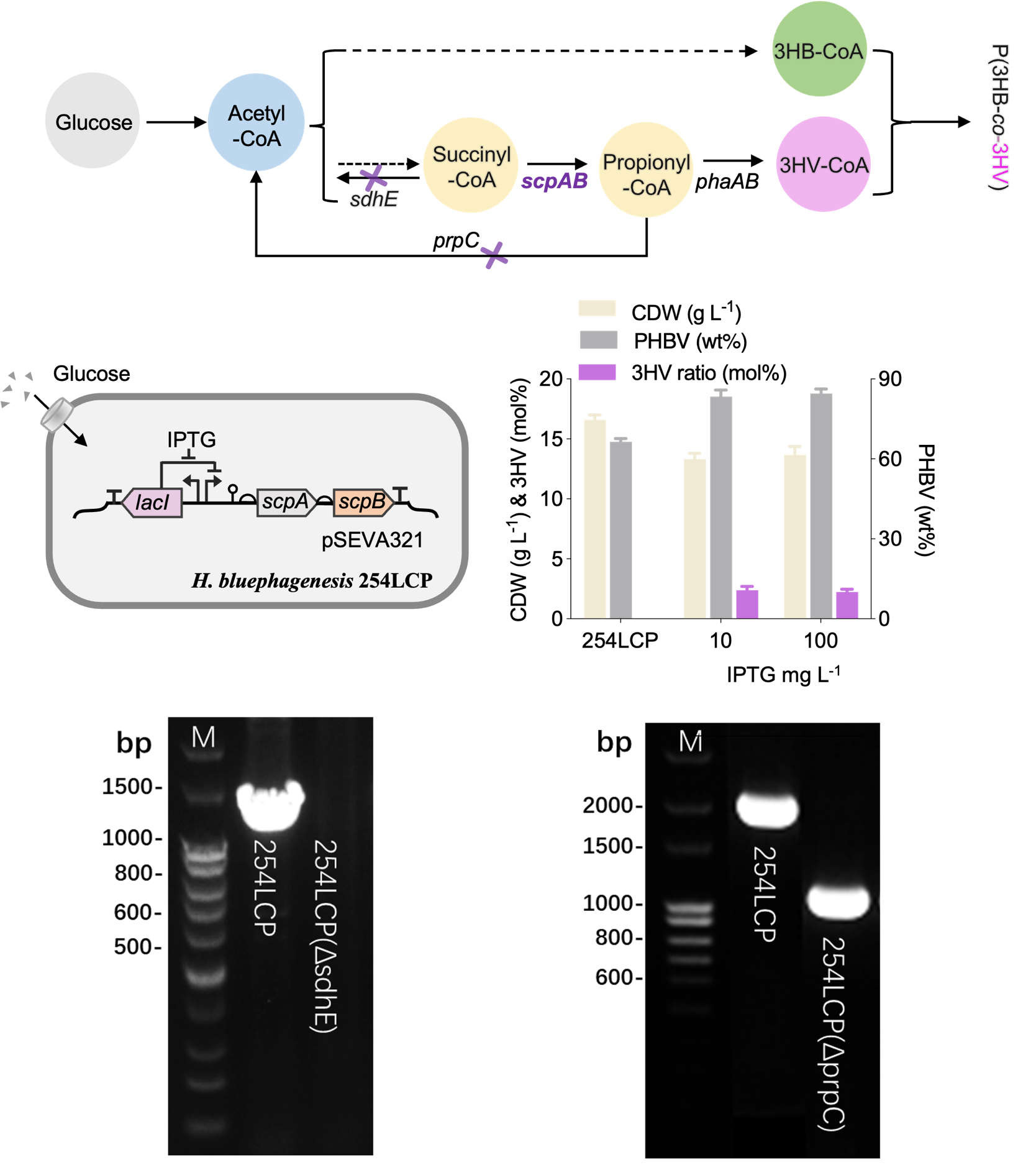
**

**Sup. Fig. 3 Metabolic engineering of *H. bluephagenesis* for synthesis of PHBV.**

a) The engineered PHBV *de novo* synthesis pathway. Deletion on *sdhE* and *prpC* genes to increase the molar ratio of 3HV in PHBV. b) Schematic diagram of the *scpAB* expression under the inducible P_mmp1_ promoter in *H. bluephagenesis* 254LCP*.* The expression level of *scpA and scpB* can be controlled by the concentration of IPTG. c) Shake-flask studies for PHBV production by *H. bluephagenesis* 254LCP and its derivatives, expressing *scpAB* with an inducible promoter, induced by IPTG with different concentrations. d) The engineered PHBV *de novo* synthesis pathway. b-c) Verification of the knockout of the *sdhE* and *prpC* genes by PCR.

**
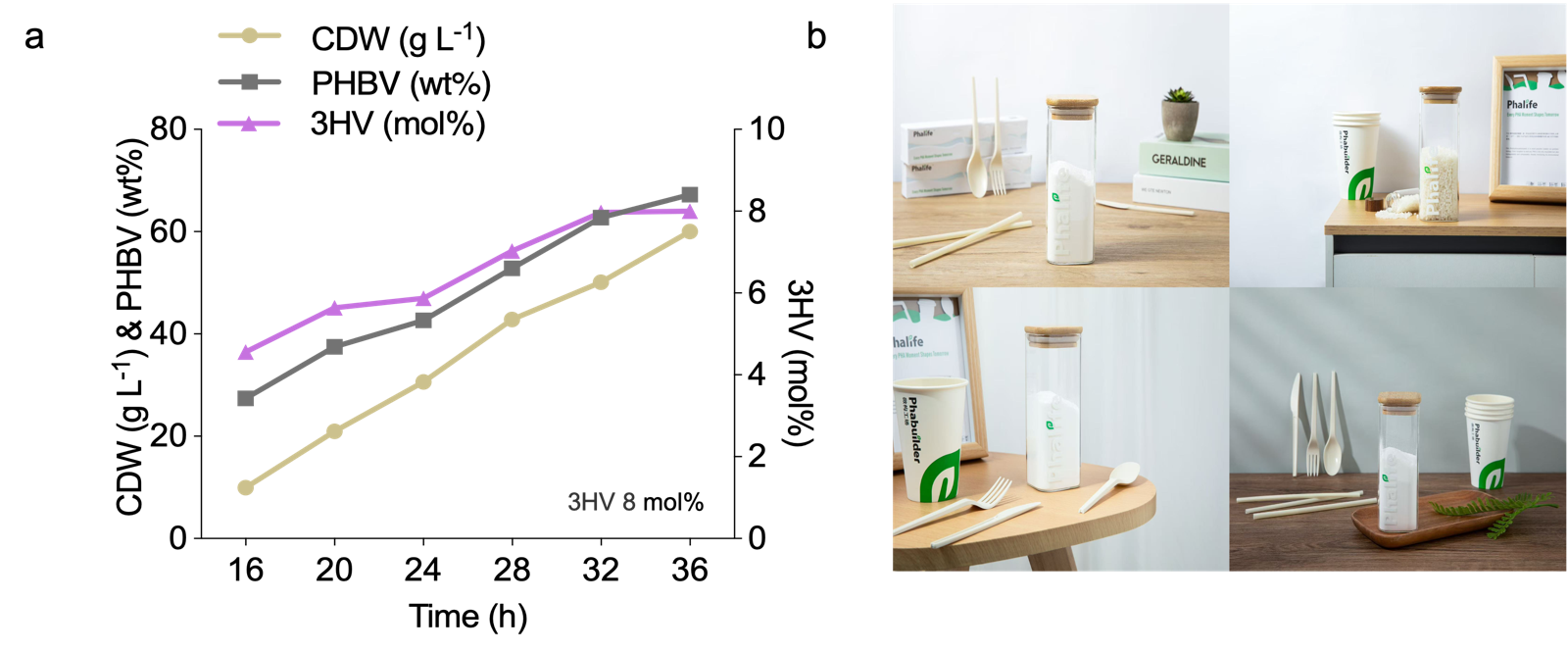
**

**Sup. Fig. 4 Relation of valerate concentration with 3HV molar ratio of PHBV formed in 7 L bioreactor and PHBV-based products.**

1. Precision control of PHBV biosynthesis was achieved by sodium valerate feeding based on the linear correlation between its concentration and 3HV molar ratio (Fig. 4i), targeting 8.8 mol% 3HV incorporation. In 7-L culture of *H. bluephagenesis*, the experimentally obtained 3HV molar ratio, cell dry weight (CDW) and PHBV content closely matched the predicted values. b) PHBV-based products made of PHA biosynthesized by *H. bluephagenesis*.

**Supplementary Table 1. Genes, plasmids and strains used in this study.**

| **Genes / Plasmids / Strains** | **Description** | **References / Sources** |
| --- | --- | --- |
| **Genes** |  |  |
| *scpA* | Methylmalonyl-CoA mutase of *Escherichia coli* | [2] |
| *scpB* | Methylmalonyl-CoA decarboxylase of *Escherichia* *coli* | [2] |
| *prpC* | Endogenous 2-methylcitrate synthase of *Halomonas bluephagenesis* TD01. | [3] |
| *sdhE* | Endogenous succinate dehydrogenase assembly factor 2 of *H. bluephagenesis* TD01. | This study |
| *phaJ_TD_* | Endogenous enoyl-CoA-hydratase of *H. bluephagenesis* TD01. | [3] |
| *fadE* | Endogenous acyl-CoA dehydrogenase of *H. bluephagenesis* TD01. | [4] |
| *fadB* | Endogenous enoyl-CoA hydratase of *H. bluephagenesis* TD01. | [4] |
| *fadA* | Endogenous 3-ketoacyl-CoA thiolase of *H. bluephagenesis* TD01. | [4] |
| *phaA* | Endogenous 3-ketothiolase of *H. bluephagenesis* TD01. | This study |
| *phaB* | Endogenous NADH-dependent acetoacetyl-CoA reductase of *H. bluephagenesis* TD01. | This study |
| **Plasmids** |  |  |
| pSEVA321 | A standardized European vector, *trfA* replication origin, *oriT*, Cm^R^. | [5] |
| p321-P_mmp1_-*scpAB* | pSEVA321 derivative, inducible expression of *scpA* and *scpB* controlled by the external addition of IPTG. | This study |
| pQ08-cas9 | pSEVA321 derivative carrying the *Streptococcus pyogenes* Cas9 expression module, Cm^R^. | [6] |
| pSEVA341-sgRNA | pSEVA341 derivative carrying an sgRNA expression module for gene editing. | [6] |
| pHbPBC-scpAB | pHbPBC derivative, *scpA* and *scpB genes driven by* P*_porin_*_203_ and P*_porin_*_278_, respectively. | This study |
| **Strains** |  |  |
| *E. coli* S17-1 | A vector donor strain used for conjugation carries the *tra* genes from plasmid RP4 on the chromosome. | [7] |
| *H. bluephagenesis* |  |  |
| TD01 | Wild-type strain isolated from Aydingol Lake in Xinjiang Province, China. | [3] |
| 254LCP | TD01 derivative with *phaP1, LpxL*, and *LpxM.* | [8] |
| GZ03 | 254LCP derivative with *sdhE* deletion and *scpAB* operon overexpression. | This study |
| GZ04 | 254LCP derivative with *prpC* deletion and *scpAB* operon overexpression. | This study |
| GZ05 | 254LCP derivative with *sdhE* and *prpC* deletion and *scpAB* operon overexpression. | This study，[1] |
| GZ06 | GZ05 derivative with *mreB* deletion with the sspB-ssrA protein degradation system. | This study |
|  |  |  |

**Supplementary Table 2. The DNA sequences of target genes.**

| **No.** | **Genes** | **Sequence** |
| --- | --- | --- |
| 1 | *prpC* | ttagtcacgcgcttcgatgggcacccactcgctcttctcaggaccagtgtagtcggcgctggggcgaataatgcggttattggcgcgctgctcgaatacgtgggctgcccagcctgtcaaacgggacatcacgaagatcggcgtaaacagcttggtcggaatatccatgaagtggtaagcactggcatggaagaagtccgcgttgcagaatagtttcttctcacgccacatgacttcttcacagcgcacagaaacggggtaaagcacgctgtcgcctacgtcatcggcaagcttctgcgaccactctttgatgatttcattacgcgggtcagactcgcggtaaatcgcatggccaaagcccatgatcttctctttgcgctcaagcataccgagcatttcgcgctcggcttcttccggcgatgcccagttctcgatcattgccatggccgcttcattagcaccgccgtgcagcgggccacgtagtgaaccaatcgcaccggtgacacaagagtgcatgtcagaaagcgtcgaggcgcaaacccgcgccgtaaacgttgaggcattgaactcatgctcggcgtatagaatcaacgatacgttcattacccgcgcatgtaactcagaagcaggctcaccgcgcagcatatgtaggaagtggccgcctacagaggcatcgtcagtttcggtatcaatgcgaacgccgtcgtggctgaaacggtaccagtagcaaataatcgaaggcagcaccgctaacaggcgatcggaaacatcctgctgctggtcaaagctctcttcagtttccaggttacccagcatggaggtaccggtgcgcataacatccatcggatgcgcatctttgggaatttgctccagcacggatttcagggcatcgggcaaaccacgcagcccctttagcttggtgatgtagccatcaagctcggcttggttaggcagcttgcctttcagtaacaaatacgcgacttcttcaaactttgccttctcggccaactctttaatatcaaaaccacggtaggttaatccggaacccgttttacccaccgtacacagcgctgtggtaccagcgctttgtccacggagtcctgcgctgttttgcggtttatcagccat |
| 2 | *sdhE* | ttgaacgacgatacttcccctgcagccatattgcgtaaacggctttattggcactctcgccgtggcatgtgggagcttgacctgctgctgattccgtttcttgagcaccgctttgatgagctgagtgaagatgaacagttagcctatcagcgtttaatcgaggaagaggatcaggatctctttggatggctgatgcgtcgtgaatggcccgaggagccatcgttaaagcgcatcgtgcagatgattgtagagcatgcagaaaataccgataactctgcttatcgtacgctctaa |

**Supplementary Table 3. Primers used in this study.**

| **Primers** | **Sequence** |
| --- | --- |
| F24 | AGCGGATAACAATTTCACACAGGA |
| *R24* | CGCCAGGGTTTTCCCAGTCACGAC |
| PscpAB-F | TAATTTCGTTGGTCATTAAGTCGTGACTGGGAAAACCTGGC |
| PscpAB-R | ACTCCTGCACGTTAGACATCTAGTATTTCTCCTCTTTCTCTAG |
| scpAB-F | AGAGAAAGAGGAGAAATACTAGATGTCTAACGTGCAGGAGTG |
| scpAB-R | GGGTTTTCCCAGTCACGACTTAATGACCAACGAAATTAGGTTTACGTT |
| *Pporin278-F* | TTCACTGGAATCCCAGTATTAAATTTGACCTGCGAGCA |
| *Pporin278-R* | TGCTCGCAGGTCAAATTTAATACTGGGATTCCAGTGAA |
| *pPhaCABLib-F* | GTGAATGCCGACCAGGTT |
| *pPorinRiboJ-R* | ACTCTTAAACAAAATTATTTGTAGAGGCTGTTT |

**Supplementary references**

[1] K. Ren, Y. Zhao, G. Q. Chen, X. Ao and Q. Wu. Construction of a Stable Expression System Based on the Endogenous *hbpB/hbpC* Toxin-Antitoxin System of *Halomonas bluephagenesis*. ACS Synth Biol 2024;13:61–67. <https://doi.org/10.1021/acssynbio.3c00622>

[2] Y. Chen, X.Y. Chen, H.T. Du, X. Zhang, Y.M. Ma, J.C. Chen, J.W. Ye, X.R. Jiang and G.Q. Chen. Chromosome engineering of the TCA cycle in *Halomonas bluephagenesis* for production of copolymers of 3-hydroxybutyrate and 3-hydroxyvalerate (PHBV). Metab Eng 2019;54:69–82. <https://doi.org/10.1016/j.ymben.2019.03.006>

[3] D. Tan, Y. S. Xue, G. Aibaidula and G. Q. Chen. Unsterile and continuous production of polyhydroxybutyrate by *Halomonas* TD01. Bioresour Technol 2011;102:8130–6. <https://doi.org/10.1016/j.biortech.2011.05.068>

[4] H. Wang, J.W. Ye, X. Chen, Y. Yuan, J. Shi, X. Liu, F. Yang, Y. Ma, J.C. Chen, F. Wu, Y. Lan, Q. Wu, Y. Tong and G.Q. Chen. Production of PHA Copolymers consisting of 3-Hydroxybutyrate and 3-Hydroxyhexanoate (PHBHHx) by Recombinant *Halomonas bluephagenesis*. Chem Eng J 2023;466:143261. <https://doi.org/10.1016/j.cej.2023.143261>

[5] R. Silva-Rocha, E. Martínez-García, B. Calles, M. Chavarría, A. Arce-Rodríguez, A. de Las Heras, A. D. Páez-Espino, G. Durante-Rodríguez, J. Kim, P. I. Nikel, R. Platero and V. de Lorenzo. The Standard European Vector Architecture (SEVA): a coherent platform for the analysis and deployment of complex prokaryotic phenotypes. Nucleic Acids Res 2013;41:D666–75. <https://doi.org/10.1093/nar/gks1119>

[6] Q. Qin, C. Ling, Y. Zhao, T. Yang, J. Yin, Y. Guo and G. Q. Chen. *CRISPR/Cas9* editing genome of extremophile *Halomonas spp*. Metab Eng 2018;47:219–229. <https://doi.org/10.1016/j.ymben.2018.03.018>

[7] R. Simon, U. Priefer and A. Pühler. A Broad Host Range Mobilization System for In Vivo Genetic Engineering: Transposon Mutagenesis in Gram Negative Bacteria. Bio/Technology 1983;1:784–791. <https://doi.org/10.1038/nbt1183-784>

[8] L. P. Yu, X. Yan, X. Zhang, X. B. Chen, Q. Wu, X. R. Jiang and G. Q. Chen. Biosynthesis of functional polyhydroxyalkanoates by engineered *Halomonas bluephagenesis*. Metab Eng 2020;59:119–130. <https://doi.org/10.1016/j.ymben.2020.02.005>
